# Single-port laparoscopic appendectomy using a needle-type grasping forceps for selective adult patients with acute uncomplicated appendicitis

**DOI:** 10.1093/jscr/rjab557

**Published:** 2022-01-14

**Authors:** Yang Chen, Yanjie Liu, Shigang Guo, Jieqing Yuan, Xiaojun Li

## Abstract

Laparoscopic appendectomy has been performed by surgeons all over the world with the advantages of minimal injury. However, conventional multiple ports procedure still has room for improvement to further reduce surgical stress. We present a novel technique of single-port laparoscopic appendectomy using a needle-type grasping forceps (SLAN) for the treatment of uncomplicated appendicitis in adults, which produces just a 1 cm traumbilical incision. Fourteen adult patients underwent this technique without any complications. Many advantages were observed, including minimal surgical trauma, less pain, faster recovery and unobviousable scars. In conclusion, SLAN provides a new choice of minimal invasive procedure for surgeons to treat adult patients with acute uncomplicated appendicitis.

## INTRODUCTION

Acute appendicitis in adults is a common acute abdomen worldwide, and surgical resection is the main treatment. In 2019, we performed a novel technique of single-port laparoscopic appendectomy using a needle-type grasping forceps (SLAN) for uncomplicated appendicitis in children, and obtained satisfactory clinical outcomes, including minimal surgical trauma, less pain, faster recovery and fewer complications [1]. However, the application of SLAN in adults remains difficult and controversial, and surgical outcomes should be confirmed by clinical practice. The objective of this study was to describe the technical details of SLAN and to evaluate its clinical outcomes in selective adult patients with uncomplicated appendicitis.

## SURGICAL TECHNIQUE

**Figure 1:**
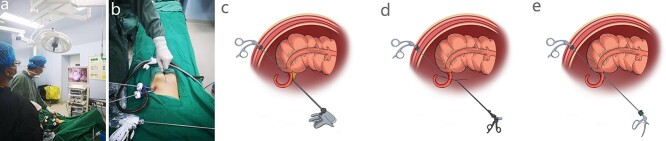
The key procedure of SLAN. (**a**) Position of surgeon and assistant. (**b**) Position of surgical incision, trocars and needle grasping forceps. (**c**) Needle grasping forceps was used to pull the appendix and ultrasonic knife was used to cutoff the mesentery. (**d**) Needle grasping forceps was used to separate and clamp the appendix. The root of the appendix should be ligated with 7–0 surgical surture. (**e**) A small Hem-o-lock clip was used to clamp the appendixroot.

An empty bladder and >6 h fasting before surgery was necessary for the patient but insertion of a gastric tube or urinary catheter was unnecessary. Under general anesthesia, the laparoscopic display was placed on the right side of the patient, who was in the supine position. The surgeon stood on the lower left of the patient, while the assistant stood on the upper left. After conventional disinfection, a pneumoperitoneum tube, 5-mm 30°-laparoscopic lens, ultrasonic scalpel and suction device were prepared (Fig. 1a).

**Figure 2:**
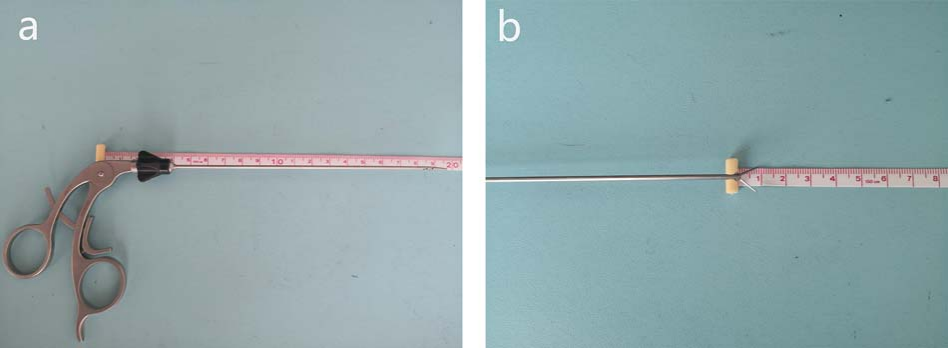
(**a**) The total length of the needle-type grasping forceps is about 15.5 cm. (**b**) The tip of the needle-type grasping forceps is about 1 cm.

A curved incision of 1 cm length was made at the lower edge of the umbilical ring, and abdominal cavity was exposed according to the open technique. The disposable bag was placed in the right upper abdominal cavity firstly to avoid affecting the exploration and operation. Two 5-mm disposable trocars were placed in the abdominal cavity; one was used for laparoscopic observation whereas the other was used as the main procedural port (Fig. 1b). Simultaneously, CO_2_ pneumoperitoneum was established, whose pressure was maintained at about 12 mm Hg. When the diseased appendix was found by laparoscopic exploration, the patient’s posture was changed to the posture of head down- foot height 30°-left side tilt 30°, and a 50-ml syringe needle pierced the abdominal wall at the McBurney site, where the needle-type grasping forceps was pierced into the abdominal cavity (Fig. 2). After the terminal ileum, the triangular ligament and cecum were identified, the mesentery of the appendix was dissociated to the root (Fig. 1c), and two 7–0 surgical sutures were ligatured to ‘slim’ the root (Fig. 1d), and a minimal Hem-o-lock clip was used to close the proximal root of the appendix (Fig. 1e). Another 7–0 surgical suture was ligated at the distal end of the appendix. The appendix was cutoff at 3–5 mm distance from the root. A one-sixteenth Iodophors gauze was placed into the abdominal cavity through the main procedural trocar, to disinfect the needle-type grasping forceps, and wipe or suck up the ascites. After the integrity of the gauze, instruments, and especially the needle-type grasping forceps was checked, and the McBurney site puncture hole was checked for absence of bleeding, the 7–0 surgical suture of the distal appendix was pulled out by laparoscopic separation forceps through the main procedural trocar. The CO_2_ pneumoperitoneum was released, two 5-mm umbilical trocars were removed, and the 7–0 surgical suture was fixed to index the appendix. A 10-mm disposable trocar was placed into the umbilical incision, and the suture was clamped with laparoscopic separation forceps, and the appendix was removed through the 10-mm disposable trocar to avoid contact with the incision. After the umbilical port was checked for absence of bleeding, the umbilical incision was sutured by a 3–0 absorbable suture.

## DISCUSSION

Acute appendicitis is a common type of acute abdomen in adult patients and appendectomy is the main treatment [2]. Nowadays, single-port laparocscopic appendectomy has been confirmed to be a feasible and safe alternative to standard laparoscopy [3–5]. The objective of SLAN is to further reduce the surgical stress by minimal incision along with several advantages: (i) it was assisted by the needle-type grasping forceps, meanwhile traditional laparoscopic surgical instruments were also used, and conventional surgical procedures and principles were followed, which can result in a short learning curve; (ii) just a 1 cm traumbilical incision can reduce patients’ surgical stress and enhance recovery process, which meets the concept of Enhanced Recovery After Surgery [6, 7] and are conducive to day surgery [8]; (3) this technique was confirmed to be performed in adult patients without limit ofBMI.

**Figure 3:**
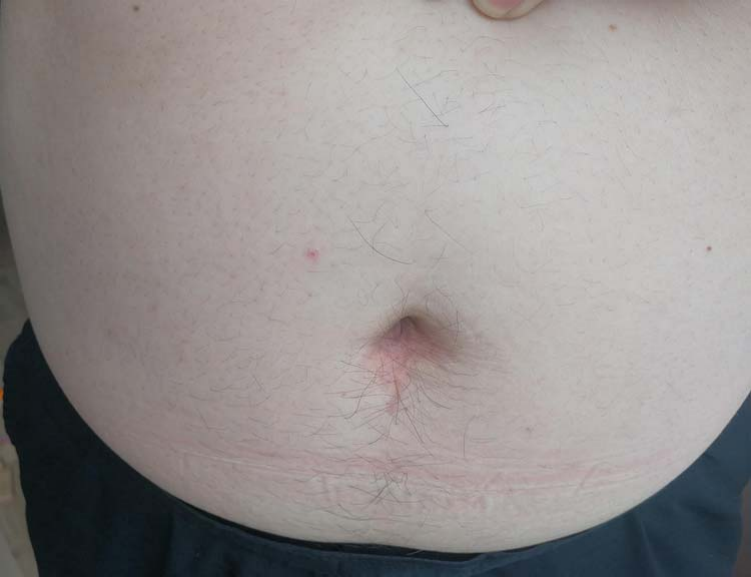
Incision healing status was observed on 9 months after surgery.

From May 2019 to April 2020, we performed 14 adult patients for acute uncomplicated appendicitis using SLAN. None of the cases were converted to open surgery or conventional laparoscopic surgery midway. The mean age was 37 years (ranged from 25 to 66 years), and the average body mass index (BMI) was 23.88 kg/m^2^ (range, 17.47–33.61 kg/m^2^). The mean operation time was 49 min (ranged from 41 to 60 min), and the mean time of first exhaust after surgery was 1.5 days (rang, 1–2 days). The average visual analogue scale (VAS) score of postoperative Day 1 was varied from 0 to 2 scores, which showed slight and tolerable pain [9]. The mean length of postoperative hospital stay was 1.7 days (ranged from 1–2 days) (Table 1). From 12 to 24 months after surgery, follow-up was accomplished by telephone, WeChat or outpatient. Neither incision infection, incision hernia, adhesive intestinal obstruction nor abdominal abscess formation was observed. No visible scars were discovered in the incision (Fig. 3), and satisfied feedback was obtained from all patients.

**Table 1:** Clinical characteristics and surgical outcomes of adult patients who underwentSLAN

Patient no.	Gender	Age (year)	BMI (kg/m^2^)	Diagnosis	Appendix diameter at base (cm)	Incision length (cm)	ASA^②^ stage	Operative time (min)	First exhaust time after surgery (d)	VAS score of POD1	SIHD	Postoperative hospital stay (d)
1	Female	50	25.58	APA	1.3	1	II	58	2	1	Grade A	2
2	Male	66	25.78	ASA^①^	1.5	1	II	50	2	2	Grade A	2
3	Female	25	24.26	APA	1.5	1	II	55	1	1	Grade A	2
4	Male	25	22.10	ASA^①^	0.6	1	II	51	1	1	Grade A	2
5	Female	29	20.58	ASA^①^	0.7	1	I	47	2	2	Grade A	2
6	Female	57	24.82	APA	0.7	1	II	50	1	1	Grade A	1
7	Female	28	19.40	APA	0.8	1	II	45	1	1	Grade A	1
8	Female	34	22.84	ASA^①^	0.6	1	II	45	2	1	Grade A	2
9	Male	55	24.65	APA	0.8	1	II	51	1	2	Grade A	2
10	Male	33	33.14	APA	0.75	1	II	60	2	2	Grade A	2
11	Female	25	20.20	APA	0.8	1	II	41	2	0	Grade A	2
12	Female	37	19.95	ASA^①^	0.8	1	I	45	2	2	Grade A	2
13	Male	29	17.47	APA	1.5	1	II	45	1	2	Grade A	1
14	Female	27	33.61	ASA^①^	0.8	1	II	50	1	1	Grade A	1

BMI: body mass index.

ASA^①^: acute simplex appendicitis.

APA: acute purulent appendicitis.

ASA^②^: American Society of Anesthesiologists.

VAS: visual analogue scale, score range 0–3 means slight and tolerablepain.

POD1: postoperative Day 1.

SIHD: surgical incision healing grades.

Grade A: surgical incision healing excellent, without any side effects.

Nonetheless, careful selection of adult cases is still important. A comprehensive medical history, physical examination and laboratory tests should be completed before surgery. SLAN should not be performed in patients with a high suspicion of appendiceal perforation and formation of abscesses around the appendix, a large number ascites, severe adhesions, or other complex conditions, or in patients who need abdominal drainage tubes. In addition, the principle of surgical asepsis should not be ignored. For example, the tip of the needle grasping forceps must be disinfected before being pulled out, and the incision should be protected without any contact when the diseased appendix is removed. Although there are many options for adult appendectomy, we offer clinicians a new strategy to further improve the cosmetic outcome.

## CONCLUSION

SLAN is proved to be a novel and simple procedure which can be safely performed in adult patients with uncomplicated appendicitis. Further research with large sample size and long-term follow-up results should be conducted.

## CONFLICT OF INTEREST STATEMENT

The authors declare no conflicts of interest in association with the present study.

## FUNDING

Natural Science Foundation of Liaoning Province, China (2019-ZD-0902).
